# Primary breast double-hit lymphoma management and outcomes: a real-world multicentre experience

**DOI:** 10.1186/s12935-021-02198-y

**Published:** 2021-09-17

**Authors:** Tingting Zhang, Yuanfeng Zhang, Hairong Fei, Xue Shi, Liang Wang, Peijun Wang, Jie Yu, Yuyan Shen, Sizhou Feng

**Affiliations:** 1 Haematopoietic Stem Cell Transplantation Centre, State Key Laboratory of Experimental Hematology, National Clinical Research centre for Blood Diseases, Institute of Hematology and Blood Diseases Hospital, Chinese Academy of Medical Sciences and Peking Union Medical College, No. 288 Nanjing Road, Tianjin, 300020 China; 2 Department of Haematology, The Affiliated Yantai Yuhuangding Hospital of Qingdao University, Yantai, 264000 Shandong China; 3 Department of Haematology, The Affiliated Hospital of Qingdao University, Qingdao, 266003 Shandong China; 4 Department of Haematology, Shengli Oilfield Central Hospital, Dongying, 257000 China; 5 Department of Haematology, Qingdao Centre Hospital, Qingdao, 266042 Shandong China; 6 Department of Haematology, Weihai Municipal Hospital, Weihai, 264200 Shandong China

**Keywords:** Primary breast lymphoma, Double-hit lymphoma, High-grade B-cell lymphomas, Therapy, Relapse, Prognosis

## Abstract

**Background:**

Primary breast double-hit lymphoma (PB-DHL) is a rare, highly aggressive malignancy that poses challenges regarding accurate diagnosis and selecting optimal treatment regimens.

**Methods:**

We retrospectively reviewed 48 cases of patients diagnosed with PB-DHL in six academic centres between June 2014 and June 2020 in China. Study-specific data were recorded, including treatment options, therapeutic evaluation, prognostic factors and relapse patterns, and the overall survival (OS) and progression-free survival (PFS) were evaluated.

**Results:**

In total, 48 patients were enrolled, with 14 patients treated with DA-EPOCH-R/MA (rituximab, dose-adjusted etoposide, prednisone, vincristine, cyclophosphamide, doxorubicin, alternating with high-dose methotrexate and cytarabine), 18 patients treated with DA-EPOCH-R (rituximab, dose-adjusted etoposide, prednisone, vincristine, cyclophosphamide, doxorubicin), and 16 patients treated with R-HyperCVAD (rituximab, hyperfractionated cyclophosphamide, vincristine, doxorubicin, dexamethasone, alternating with cytarabine plus methotrexate). The overall 5-year OS and PFS rates were 41.7% (95% confidence interval [CI], 27.6–56.8%) and 37.5% (95% CI, 24.0–52.6%), respectively. Of the three treatment regimens, the 5-year OS was higher in DA-EPOCH-R/MA group than in the DA-EPOCH-R or R-HyperCVAD subgroups (57.1% vs. 38.9% vs. 31.3%; P = 0.016), as was the 5-year PFS (50.0% vs. 38.9% vs. 25.0%; P = 0.035). Autologous stem cell transplantation (ASCT) prolonged the OS and PFS compared with non-ASCT patients (5-year OS: 72.2% vs. 23.3%; P < 0.001; 5-year PFS: 72.2% vs. 16.7 %, P < 0.001). Multivariate analysis identified tumour size, risk stratification, treatment with DA-EPOCH-R/MA, breast irradiation, and ASCT as significant prognostic factors.

**Conclusions:**

DA-EPOCH-R/MA is a promising regimen for PB-DHL, and breast irradiation yields complementary benefits for prognosis. ASCT significantly decreased disease relapse, providing a potential curative PB-DHL intervention and justifying ASCT as first-line therapy for young patients. More effective treatment strategies for PB-DHL patients remain encouraging.

**Supplementary Information:**

The online version contains supplementary material available at 10.1186/s12935-021-02198-y.

## Introduction

Primary breast lymphoma (PBL) is a rare subtype of breast malignancy, accounting for 1% of non-Hodgkin lymphomas and less than 3% of extra-nodal lymphomas [1–3]. The definition of PBL was proposed by Wiseman and Liao [4] in 1972, in which breast tissue was infiltrated by lymphoma cells with or without regional lymph node involvement. More than 95% of all diagnosed breast lymphoid malignancies are expected to be B-cell non-Hodgkin lymphomas, such as follicular lymphoma and diffuse large B-cell lymphoma (DLBCL) [5, 6]. The typical presentation is a unilateral painless breast mass in middle-aged women [2, 7]. However, accumulating evidence indicates that the morbidity continues to increase for younger women with increasing incidence over the last four decades [8]. Consequently, more attention is required. Double-hit lymphoma is high-grade B-cell lymphoma (HGBL) with MYC and B-Cell Leukaemia/Lymphoma (BCL)2 or BCL6 gene translocations detected by fluorescence in situ hybridization (FISH) or standard cytogenetics according to the National Comprehensive Cancer Network guidelines [9], representing a rare subtype with typical resistance to conventional therapy [10].

There are no accepted guidelines for standardised primary breast double-hit lymphoma (PB-DHL) treatment strategies, although combined chemotherapy and radiotherapy are presently the first-line therapeutics [3, 11, 12]. Most studies still recommend the routine use of prophylactic central nervous system (CNS) therapy. However, the optimal approach for CNS prophylaxis remains unclear, and the CNS recurrence rate varies widely among studies [12, 13]. In light of the scarcity of data on Chinese patients, the disease is poorly understood, and no large studies have reported on PB-DHL treatment, making an accurate diagnosis and disease management a challenge [12]. The multicentre North-China collaboration was initiated to analyse the clinicopathologic features, explore effective curative options, and facilitate the development of effective prophylactic CNS strategies.

## Patients and methods

**Fig. 1:**
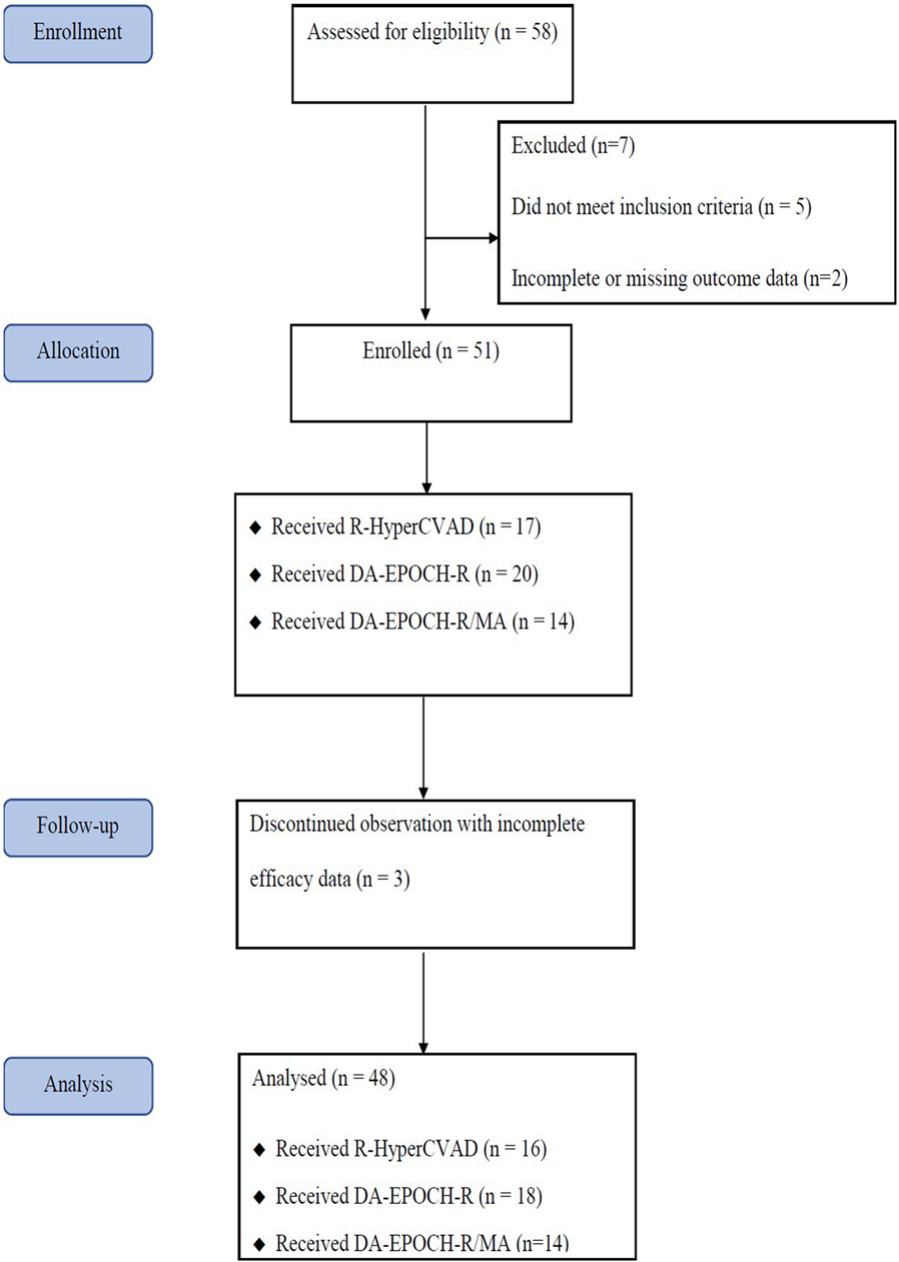
Flow diagram to identify patients with PB-DHL. * PB-DHL* primary breast double-hit lymphoma, *R-HyperCVAD* rituximab, hyperfractionated cyclophosphamide, vincristine, doxorubicin and dexamethasone, alternating with cytarabine plus methotrexate, *DA-EPOCH-R* rituximab, dose-adjusted etoposide, prednisone, vincristine, cyclophosphamide, doxorubicin, *DA-EPOCH-R/MA* rituximab, dose-adjusted etoposide, prednisone, vincristine, cyclophosphamide, doxorubicin, alternating with high-dose methotrexate and cytarabine

The clinical data of consecutive patients between June 2014 and June 2020 were derived from six hospital databases in China. All diagnostic materials were recategorised and met the 2016 World Health Organization’s (WHO) classification of lymphoid neoplasms [14]. The patients’ pathological sections were rechecked by two senior pathologists. The inclusion criteria were: breast was the definite primary site, patients with HGBL with MYC and BCL2 or BCL6 gene translocations, and patients diagnosed at 60 years old or younger. Patients were excluded if they had a primary site that was difficult to confirm, had breast involvement secondary to systemic disease, had a prior diagnosis of haematologic malignancy, were histologically confirmed as non-HGBL, or had incomplete or missing outcome data. Based on the PBL definition, patients with bilateral breast involvement at the first diagnosis were classified as stage IV in this study. The collected data included clinicopathological results, the Ann Arbor staging classification, treatment strategies, adverse effects, and survival. After PB-DHL was diagnosed, computed tomography or positron emission tomography scans were performed for staging purposes. The response was evaluated by the time of completion or the initial treatment interim based on the revised version of the International Working Group in 2007 [15]. Toxic effects and haematological and non-haematological toxicity profiles were evaluated and graded based on the WHO’s Common Toxicity Criteria (http://ctep.cancer.gov, version 3.0). All patients were followed up until 1 July 2020. Informed consent was obtained from the patients or guardians of the study participants. All procedures followed the ethical standards of the Institutional Research Committee, and this study was approved by an appropriate ethics committee. The process of screening and identifying eligible patients is presented in Fig. 1.

### Statistical analyses

The primary endpoints were overall survival (OS) and progression-free survival (PFS). The secondary endpoints were toxic effects and overall response rate (ORR), including complete remission (CR) and partial remission (PR). The baseline characteristics across the disease subgroups were compared using the Fisher’s exact test. Survival was evaluated by Kaplan–Meier survival analysis, and any differences in survival were estimated using the log-rank test. Prognostic factors (P < 0.1) in univariate analysis were subjected to the Cox proportional-hazards model for multivariate survival analysis to determine the simultaneous impact of prognostic factors on survival. Interactions between prognostic factors were also assessed using the Cox proportional-hazards model. OS was determined from the date of diagnosis until the death date or last follow-up. PFS was calculated from the date of diagnosis to the date of the first progression, relapse, or death of any cause. All statistical analyses were performed using GraphPad Prism software version 8.0 (GraphPad Software Inc., San Diego, CA, USA) and R version 3.4.3 (R Foundation for Statistical Computing, Vienna, Austria). Statistical significance was set as a two-tailed P-value < 0.05.

## Results

### Baseline characteristics

Totally 48 patients were enrolled in this study. The baseline characteristics of the 48 enrolled patients are presented in Table 1. The median age was 48 years (range, 24–61 years). A painless unilateral breast mass was present in 38 patients (79.2%), and the left breast was involved more often than the right (45.8% vs. 33.3 %). The median tumour size was 2.6 cm (range, 0.9–11.5 cm); 17 patients had a tumour > 5 cm, and one had a tumour > 10 cm. There were 13 patients classified as Ann Arbor stage IE, 21 classified as IIE, and 14 classified as IV. Risk stratification was investigated based on age-adjusted International Prognostic Index scores; 27 patients fell into the low- and low-intermediate risk categories and 21 patients into the high-intermediate and high-risk categories. According to the Hans’ algorithm, patients were divided into two types: the germinal center B-cell (GCB) type (n = 40, CD10 + or CD10-BCL6 + MUM1-) and non-GCB type (n = 8, CD10-BCL6- or CD10-BCL6 + MUM1+), by semi-quantitatively scoring the fraction of tumor cells stained using a 30% threshold [16]. Based on the cell of origin, 40 patients (83.3 %) were of the GCB type. Immunohistochemistry (IHC) was performed using formalin-fixed paraffin-embedded breast tissue biopsies prepared at the time of diagnosis. All cases were positive for C-MYC protein. 43 cases were both positive for BCL2 and BCL6 protein. 3 cases were only positive for BCL2 and 2 cases were only positive for BCL6. Chromosomal abnormalities were observed in 12 of 48 patients. 38 patients (79.2 %) were investigated with FISH for C-MYC and BCL2 translocations, while the remaining 10 patients were detected with FISH for C-MYC and BCL6 translocations. Next-generation sequencing (NGS)-based technologies was applied in 14 samples to identify lymphoma-specific genetic aberrations. 4 patients were found with TP53 gene mutation, 2 with BCL6 mutation, 2 with BCL2 mutation, 2 with CD79B, 2 with IGHD mutation, and the remaining cases were mutations related to unclear clinical significance. 14 patients were treated with DA-EPOCH-R/MA, 16 patients were treated with R-HyperCVAD, and 18 patients were treated with DA-EPOCH-R. Eighteen patients underwent autologous stem cell transplantation (ASCT) after the initial therapy. After a median follow-up of 47 months (range, 9.5–79 months), 28 patients died. The causes of death were infection-related (n = 7), disease progression (n = 16), second malignancy (n = 2), and non-treatment-related (n = 2), and transplant-related (n = 1).

**Table 1:** Clinical characteristics of PB-DHL patients (n = 48)

Variable	DA-EPOCH-R/MA (n, % )	DA-EPOCH-R (n, % )	R-HyperCVAD (n, % )	P value
Age				0.711
≤ 50 years	9 (64.3)	9 (50.0)	10 (62.5)	
50–60 years	5 (35.7)	9 (50.0)	6 (37.5)	
Laterality				0.965
Right	4 (28.6)	7 (38.9)	5 (31.3)	
Left	7 (50.0)	8 (44.4)	7 (43.8)	
Bilateral	3 (21.4)	3 (16.7)	4 (25.0)	
Tumour size				0.811
< 5 cm	8 (57.1)	11 (61.1)	11 (68.8)	
≥ 5 cm	6 (42.9)	7 (38.9)	5 (31.3)	
Cell of origin				0.890
GCB	11 (78.6)	15 (83.3)	14 (87.5)	
Non-GCB	3 (21.4)	3 (16.7)	2 (12.5)	
Results of FISH				0.745
MYC-BCL2	10 (71.4)	15 (83.3)	13 (81.3)	
MYC-BCL6	4 (28.6)	3 (16.7)	3 (18.8)	
C-MYC of IHC				1.000
C-MYC (+)	14 (100.0)	18 (100.0)	16 (100.0)	
BCL2 of IHC				0.521
BCL2 (+)	13 (92.9)	18 (100.0)	15 (93.8)	
BCL6 of IHC				0.767
BCL6 (+)	14 (100.0)	16 (88.9)	15 (93.8)	
CD5 of IHC				0.406
CD5 (+)	5 (35.7)	4 (22.2)	7 (43.8)	
CD10 of IHC				0.661
CD10 (+)	10 (71.4)	10 (55.6)	9 (56.3)	
P53 of IHC				0.926
P53 (+)	9 (64.3)	13 (72.2)	11 (68.8)	
Ki-67 of IHC				0.668
Ki-67 ≥ 70 %	12 (85.7)	17 (94.4)	15 (93.8)	
Chromosomal abnormality				0.700
Present	3 (21.4)	6 (33.3)	3 (18.8)	
Ann Arbor Staging				0.832
IE	4 (28.6)	6 (33.3)	3 (18.8)	
IIE	7 (50.0)	7 (38.9)	7 (43.8)	
IV	3 (21.4)	5 (27.8)	6 (37.5)	
B symptoms				0.652
Present	10 (71.4)	10 (55.6)	10 (62.5)	
LDH level				0.544
Elevated	5 (35.7)	7 (38.9)	9 (56.3)	
Risk stratification				0.665
L and L-I	9 (64.3)	9 (50.0)	9 (56.3)	
H and H-I	5 (35.7)	9 (50.0)	7 (43.8)	
ASCT				0.811
Yes	6 (42.9)	7 (38.9)	5 (31.3)	

*PB-DHL* primary breast double-hit lymphoma, *R-HyperCVAD* rituximab, hyperfractionated cyclophosphamide, vincristine, doxorubicin, dexamethasone, alternating with cytarabine plus methotrexate, *DA-EPOCH-R* rituximab, dose-adjusted etoposide, prednisone, vincristine, cyclophosphamide, doxorubicin, *DA-EPOCH-R/MA* rituximab, dose-adjusted etoposide, prednisone, vincristine, cyclophosphamide, doxorubicin, alternating with high-dose methotrexate and cytarabine, *FISH* fluorescence in situ hybridization, *IHC* immunohistochemistry, *LDH* lactate dehydrogenase, *GCB* germinal centre B-cell, *L* low risk, *L-I* low-intermediate risk *H-I* high-intermediate risk, *H* high risk, *ASCT* autologous stem cell transplantation

### Treatment and efficacy

The treatment options are summarised in Table 2, and the regimen doses are outlined in Table 3. Rituximab-containing chemical regimens were scheduled to be administered to all patients. R-HyperCVAD was used in 16 cases, DA-EPOCH-R in 18 cases, and DA-EPOCH-R/MA in 14 cases. In the R-HyperCVAD subgroup, 12 of 16 patients were treated with A and B regimens and the median cycles were 4. After the median cycles of 3 cycles, 4 patients discontinued alternating A and B cycles in the last few courses owing to poor tolerance and severe bone marrow suppression. There were nine CR cases and two PR cases, and the ORR was 68.8 %. In the DA-EPOCH-R subgroup, five of 18 patients underwent the DA-EPOCH-R regimen for 4–5 treatment cycles with the median cycles of 4, and the rest underwent 6–8 cycles with the median cycles of 7. There were eight CR cases and three PR cases, and the ORR was 61.1 %. In the DA-EPOCH-R/MA subgroup, 10 of 14 patients received 7–8 treatment cycles, and four received 4–6 cycles. There were seven CR cases and two PR cases, and the ORR was 64.3 %. More importantly, 4 patients with TP53 mutation all obtained PR even after high-dose of chemotherapy, and one of them co-occurrence with complex karyotypes relapse after ASCT.

**Table 2:** Summary of treatment

Treatment strategy	PB-DHL, N (%)
Breast irradiation	31 (64.6)
Sequence of breast irradiation	
Breast irradiation with concurrent chemotherapy	11 (22.9)
Breast irradiation following chemotherapy	20 (41.7)
Chemotherapy protocol	
R-HyperCVAD	16 (33.3)
DA-EPOCH-R	18 (37.5)
DA-EPOCH-R/MA	14 (29.2)
ASCT	18 (37.5)

*PB-DHL* primary breast double-hit lymphoma, *R-HyperCVAD* rituximab, hyperfractionated cyclophosphamide, vincristine, doxorubicin, dexamethasone, alternating with cytarabine plus methotrexate, *DA-EPOCH-R* rituximab, dose-adjusted etoposide, prednisone, vincristine, cyclophosphamide, doxorubicin, *DA-EPOCH-R/MA* rituximab, dose-adjusted etoposide, prednisone, vincristine, cyclophosphamide, doxorubicin, alternating with high-dose methotrexate and cytarabine, *ASCT* autologous stem cell transplantation

**Table 3:** Therapeutic regimens

Regimens	Dose	Days of administration
R-HyperCVAD regimen		
Cycle A		
Rituximab	375 mg/m^2^	1
Cyclophosphamide	300 mg/m^2^ every 12 h	2–4
Vincristine	1.4 mg/m^2^	4, 11
Doxorubicin	50 mg/m^2^	4
Dexamethasone	40 mg per day	1–4, 11–14
Cycle B		
Rituximab	375 mg/m^2^	1
Methotrexate	1 g/m^2^ continuous intravenous infusion for 24 h	2
Cytarabine	3 g/m^2^ every 12 h	3, 4
DA-EPOCH-R regimen ^a^		
Rituximab	375 mg/m^2^	1
Etoposide	50 mg/m^2^ continuous intravenous infusion for 24 h	2–5
Doxorubicin	10 mg/m^2^ continuous intravenous infusion for 24 h	2–5
Vincristine	0.4 mg/m^2^ continuous intravenous infusion for 24 h	2–5
Cyclophosphamide	750 mg/m^2^	6
Prednisone	60 mg/m^2^	2–6
DA-EPOCH-R/MA regimen		
DA-EPOCH-R		
Rituximab	375 mg/m^2^	1
Etoposide	50 mg/m^2^ continuous intravenous infusion for 24 h	2–5
Doxorubicin	10 mg/m^2^ continuous intravenous infusion for 24 h	2–5
Vincristine	0.4 mg/m^2^ continuous intravenous infusion for 24 h	2–5
Cyclophosphamide	750 mg/m^2^	6
Prednisone	60 mg/m^2^	2–6
Alternating with MA		
Methotrexate	3 g/m^2^	1
Cytarabine	2 g/m^2^ every 12 h	2–3
Intrathecal prophylaxis		
Methotrexate	10 mg	
Dexamethasone	10 mg	
Cytarabine	50 mg	

*PB-DHL* primary breast double-hit lymphoma, *R-HyperCVAD* rituximab, hyperfractionated cyclophosphamide, vincristine, doxorubicin, dexamethasone, alternating with cytarabine plus methotrexate, *DA-EPOCH-R* rituximab, dose-adjusted etoposide, prednisone, vincristine, cyclophosphamide, doxorubicin, *DA-EPOCH-R/MA*, rituximab, dose-adjusted etoposide, prednisone, vincristine, cyclophosphamide, doxorubicin, alternating with high-dose methotrexate and cytarabine, *MA* high-dose methotrexate and cytarabine, *IV* intravenous infusion

^a^The doses of etoposide, doxorubicin, and cyclophosphamide were adjusted according to criteria [23, 35]

ASCT was performed in 18 patients, with 6 cases in DA-EPOCH-R/MA group, 7 cases in DA-EPOCH-R group, and 5 cases in R-HyperCVAD group. Before ASCT, 13 patients achieved CR, in which 9 were first complete remission (CR1) and 4 were second complete remission (CR2). No significant differences of prognosis were found among CR1 and CR2 patients following ASCT (P > 0.05).

Breast irradiation was performed in 31 of 48 patients (64.6%) with a median dose of 30 Gy. Twenty patients received breast irradiation following chemotherapy and 11 received breast irradiation with concurrent chemotherapy. The breast radiotherapy sequence did not affect ORR (P > 0.05). None of the patients underwent contralateral breast or cranial radiotherapy.

All patients received the prophylactic CNS strategy; 26 received intrathecal chemoprophylaxis 6–8 times, and 22 received intrathecal chemoprophylaxis 4–5 times. Kaplan–Meier survival analysis showed that the intrathecal chemoprophylaxis time did not affect long-term survival. MA-containing regimen (R-HyperCVAD or DA-EPOCH-R/MA) was administered in 30 cases. Given that MA regimen has achieved CNS prophylaxis, intrathecal chemoprophylaxis wasn’t applied to patients any more during the chemotherapy of MA.

### Toxic effects

Information on toxic effects was recorded and assessed based on the WHO’s Common Toxicity Criteria; haematologic toxicity was the most common adverse effect. Grade 3/4 myelosuppression in the R-HyperCVAD subgroup was more severe than that in the DA-EPOCH-R and DA-EPOCH-R/MA subgroups (Fisher’s exact test; 75% vs. 16.7% vs. 28.6 %, respectively; P = 0.001). Other toxic effects, such as liver and kidney function damage, gastrointestinal reactions, haemorrhage, and cardiotoxicity, were mild and manageable, and the differences were insignificant. These side effects returned to normal after a short period of symptomatic and supportive treatment.

### Survival analysis

**Fig. 2:**
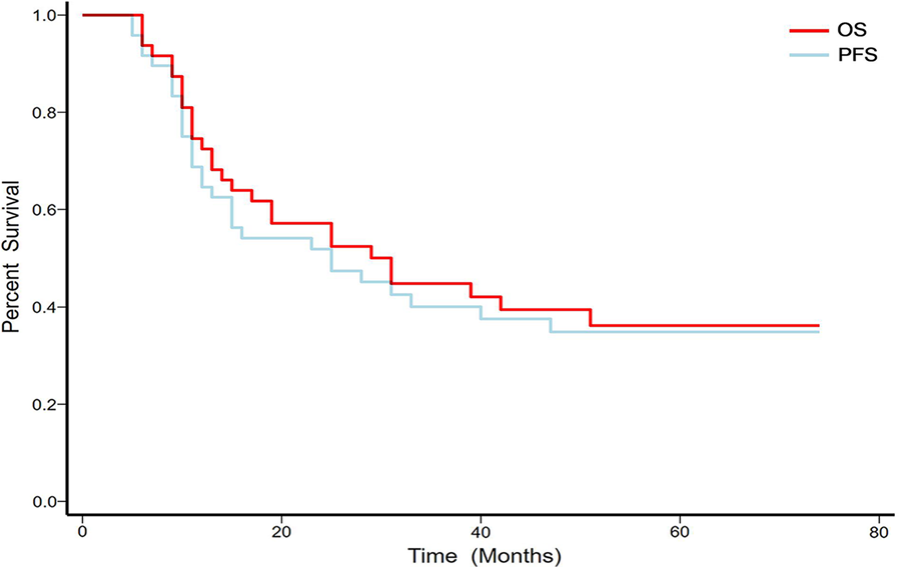
The Kaplan–Meier overall (**A**) and progression-free (**B**) survival curves

**Fig. 3:**
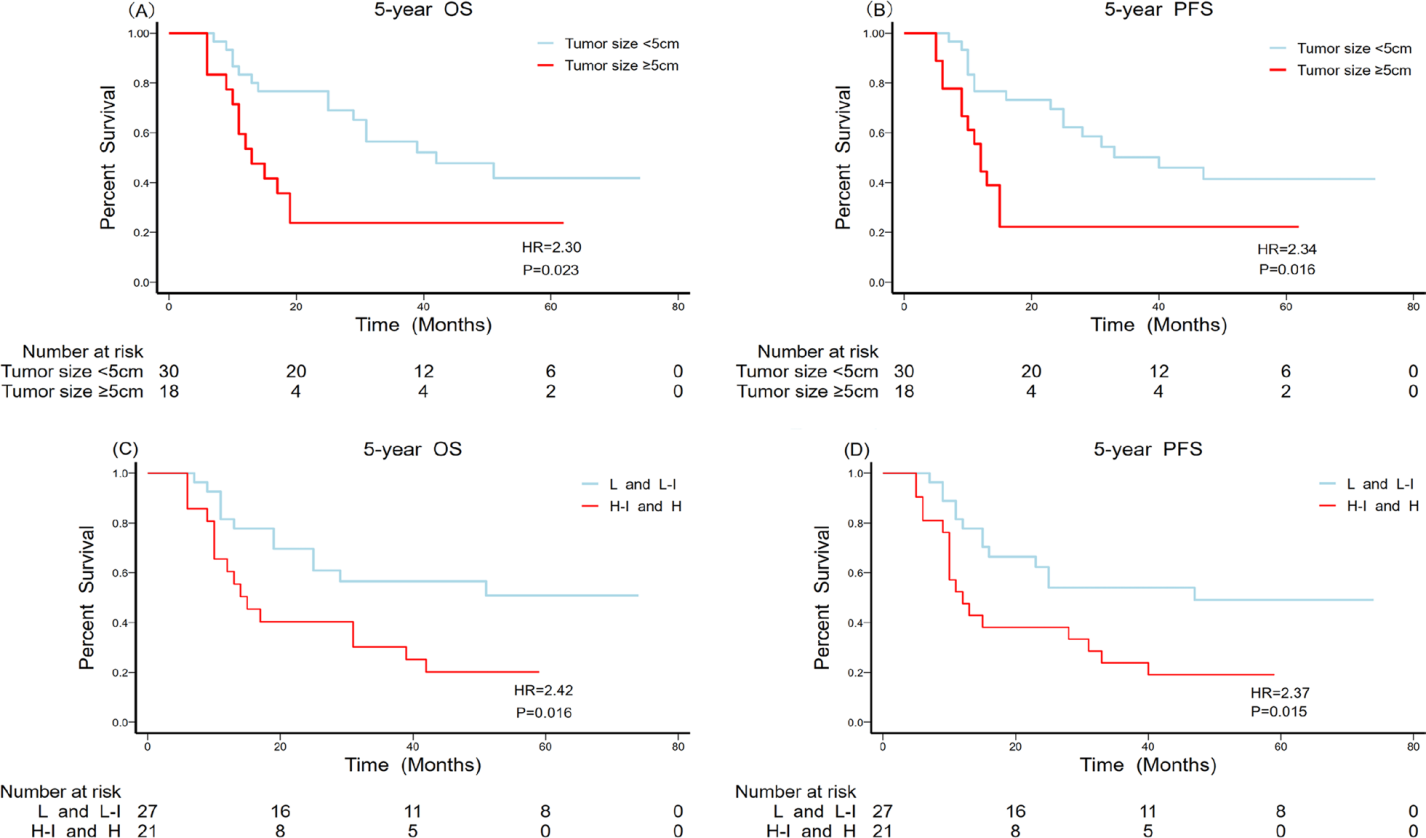
Overall and progression-free survival comparisons for patients with different tumour sizes (**A**, **B**) and risk stratifications (**C**, **D**)

**Fig. 4:**
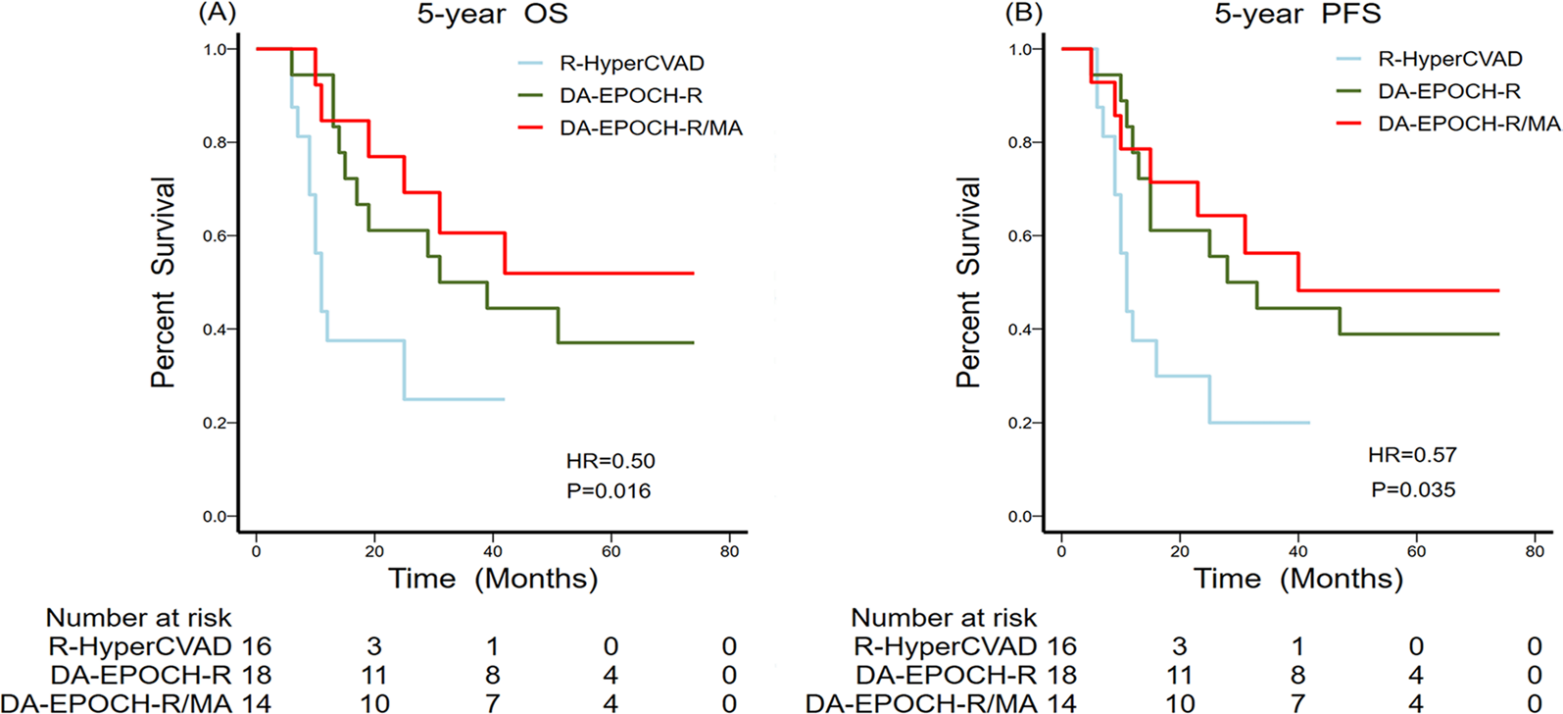
Overall (**A**) and progression-free (**B**) survival comparisons among the three treatment regimens

**Fig. 5:**
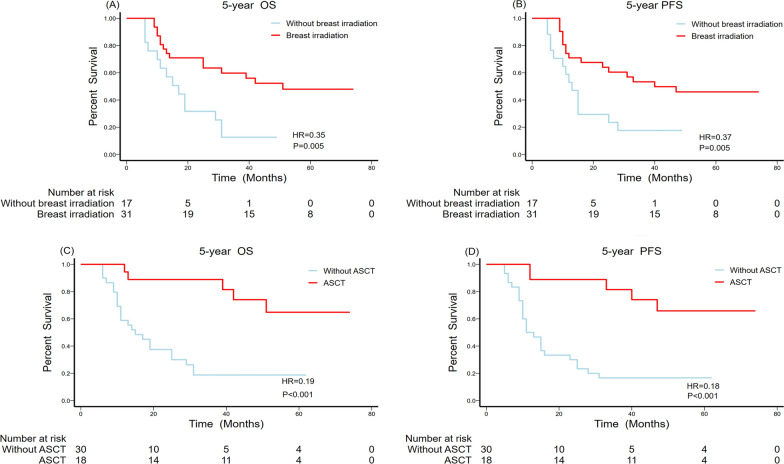
Overall and progression-free survival comparisons for breast radiotherapy (**A**, **B**) and autologous stem cell transplantation (**C**, **D**)

The univariate and multivariate analyses of prognostic factors are outlined in Table 4. The median OS was 29.0 ± 7.2 months (95% CI, 14.9–43.1 months) and the median PFS was 25.0 ± 8.5 months (95% CI, 8.3–41.7 months). As shown in Fig. 2, the overall five-year OS and PFS were 41.7% (95% CI, 27.6–56.8 %) and 37.5% (95% CI, 24.0–52.6 %), respectively. The potentially significant factors from the univariate analysis (P < 0.1) included laterality, tumour size, the Ann Arbor staging classification, risk stratification, B symptoms, chemical regimens, breast radiotherapy, and ASCT and were included in the multivariate analysis. Multivariate analysis with the Cox proportional-hazards model identified tumour size (Fig. 3A, B), risk stratification (Fig. 3C, D), treatment with DA-EPOCH-R/MA (Fig. 4), breast radiotherapy (Fig. 5A, B), and ASCT (Fig. 5C, D) as independent prognostic factors.

**Table 4:** Univariate and multivariate analysis for OS and PFS in the study cohort

Factors		Univariate analysis						Multivariate analysis					
		OS			PFS			OS			PFS		
		HR	95% CI	P-value	HR	95% CI	P-value	HR	95% CI	P-value	HR	95% CI	P-value
Age													
≤ 50 years		1.0	0.5–2.1	0.965	0.9	0.4–1.9	0.845						
50–60 years													
Laterality													
Left		0.3	0.1–0.7	0.005	0.3	0.1–0.6	0.003	3.580	0.475-26.969	0.216	2.215	0.326–15.045	0.416
Right		0.2	0.1–0.6	0.003	0.2	0.1–0.6	0.004	0.848	0.170–4.230	0.840	0.673	0.142–3.198	0.618
Bilateral													
Tumor size													
≥ 5 cm		2.3	1.1–4.9	0.030	2.3	1.1–4.8	0.022	4.771	1.624–14.020	0.004	4.517	1.643–12.416	0.003
< 5 cm													
Cell of origin													
GCB		1.1	0.4- 3.0	0.776	1.2	0.5–3.1	0.712						
Non-GCB													
Results of FISH													
MYC-BCL2		0.5	0.2–1.2	0.114	0.6	0.3–1.3	0.199						
MYC-BCL6													
BCL2 of IHC													
BCL2 +		1.3	0.2–9.4	0.809	1.4	0.2–10.3	0.743						
BCL6 of IHC													
BCL2 = 6 +		0.9	0.2–3.7	0.862	1.0	0.2–4.2	0.991						
CD5 of IHC													
CD5 +		1.8	0.8–3.7	0.144	1.6	0.7–3.2	0.240						
CD10 of IHC													
CD10 +		1.1	0.5–2.4	0.759	1.1	0.5–2.3	0.785						
P53 of IHC													
P53 +		1.4	0.6–3.3	0.452	1.6	0.7–3.7	0.295						
Ann Arbor staging													
IE		0.1	0-0.4	0.001	0.1	0-0.5	0.001	1.4	0.1–21.7	0.801	5.3	0. 4-68.7	0.203
IIE		0.3	0.2–0.8	0.010	0.4	0.2–0.8	0.009	0.9	0.1–8.2	0.903	1.9	0.2–16.3	0.558
IV													
B symptom													
Absent		0.4	0.2-1.0	0.057	0.5	0.2-1.0	0.062	0.5	0.2–1.4	0.180	0.5	0.2–1.4	0.187
Present													
LDH level													
Normal		0.7	0.3–1.5	0.405	0.9	0.4–1.8	0.693						
Elevated													
Chromosomal abnormality													
Present		0.9	0.4–2.2	0.869	0.9	0.4–2.1	0.811						
Absent													
Risk stratification													
L and L-I		0.4	0.2–0.9	0.022	0.4	0.2–0.9	0.020	0.2	0–1.0	0.050	0.2	0–0.7	0.012
H and H-I													
Breast irradiation													
Yes		0.4	0.2–0.8	0.008	0.4	0.2–0.8	0.008	0.3	0.1-1.0	0.042	0.3	0.1–0.8	0.021
No													
Chemical regimen													
R-DAEPOCH		0.4	0.2–0.9	0.036	0.4	0.2-1.0	0.047	0.1	0-0.5	0.001	0.1	0-0.4	0.001
R-DAEPOCH/MA		0.3	0.1–0.8	0.013	0.3	0.1–0.9	0.029	0.2	0.1–0.6	0.007	0.3	0.1–0.8	0.018
R-HyperCVAD													
ASCT													
Yes		0.2	0.1–0.6	0.001	0.2	0.1–0.5	< 0.001	0.1	0–0.4	0.002	0.1	0-0.3	< 0.001

*OS* overall survival, *PFS* progression-free survival, *HR* hazard ratio, *95% CI* 95% confidence interval, *GCB* germinal centre B-cel, *LDH* lactate dehydrogenase, *L* low-risk, *L-I* low-intermediate risk, *H* high-risk, *H-I* high-intermediate risk, *R-HyperCVAD* rituximab, hyperfractionated cyclophosphamide, vincristine, doxorubicin, dexamethasone, alternating with cytarabine plus methotrexate, *DA-EPOCH-R* rituximab, dose-adjusted etoposide, prednisone, vincristine, cyclophosphamide, doxorubicin, *DA-EPOCH-R/MA* rituximab, dose-adjusted etoposide, prednisone, vincristine, cyclophosphamide, doxorubicin, alternating with high-dose methotrexate and cytarabine, *ASCT* autologous stem cell transplantation

### Disease relapse

Disease relapse occurred in 18 patients; three patients without breast irradiation had contralateral breast relapse, seven patients had CNS relapse, and eight patients had lymph node relapse.

The CNS recurrence rate was 10% in patients who received the MA-containing regimen and 22.2% in patients who did not (P > 0.05). In contrast, CNS did not progress in any patient following ASCT, but 23.3% of patients who did not receive ASCT had progression. Therefore, ASCT was associated with a significantly decreased risk of early CNS progression (P = 0.036).

Contralateral breast tissue was the second most involved extranodal site. In this cohort, three patients without breast irradiation experienced contralateral breast relapse, including one with contralateral breast relapse occurring during salvage therapy. Tumour size and axillary lymph nodes were not related to the recurrence of the contralateral breast in this cohort.

## Discussion

PB-DHL is rare but deserves discussion because its biological characteristics are quite different from those of the more typical B-cell lymphoma [4]. PB-DHL typically presents in the 50 or 60 s as a painless, solitary, palpable breast lump, indistinguishable from breast cancer [5]. Previous studies identified that PB-DHL predominantly occurs in women, commonly perimenopausal women. However, some reports have included male patients [17, 18]. In this study, all cases were women, consistent with other reports.

Immunochemotherapy combined with breast radiotherapy is the currently recognised first-line therapy for PBL [2]. Research over the past decades has identified that surgical treatment offers little benefit to long-term prognosis and is no longer the mainstream of treatment [6, 13]. In this study, surgery was only employed for excisional biopsies rather than for treatment. The role of radiotherapy was clarified in clinical trials during the pre-rituximab era [19], but the benefits of radiotherapy for PB-DHL patients receiving rituximab-containing regimens remains controversial. The research indicates that immunochemotherapy alone was insufficient to achieve excellent control of local tumours, and the combination of radiotherapy and chemotherapy had the strongest benefit before ASCT. Breast irradiation also contributed to durable remission with improved OS and PFS, justifying its consideration for high-risk young patients. Relevant observations have also highlighted the beneficial effects of radiotherapy in PBL patients treated with rituximab-based regimens, which compensated for the deficiency of immunochemotherapy in the local control of residual disease or recurrence [20]. A median breast irradiation dose of 30 Gy is widely recommended [2, 3, 6].

Because there are no standard guidelines for PB-DHL [21], making an accurate diagnosis and choosing optimal regimens is challenging [22, 23]. This research showed that the efficacy benefits of the three treatment schemes were not significant, but the DA-EPOCH-R/MA group had the best survival outcomes, followed by the DA-EPOCH-R group. Although the R-HyperCVAD remission rate was slightly higher than that of the other two groups, severe bone marrow suppression might lead to treatment interruption, hampering its application. Kieron et al. [24] conducted a prospective Phase II study of DA-EPOCH-R in aggressive B-cell lymphoma with MYC rearrangement and emphasised that the DA-EPOCH-R regimen produced durable remission and could be applied for treating aggressive B-cell lymphoma. The Spanish PETHEMA group [25] conducted a Phase II study of DA-EPOCH-R in untreated patients with poor prognosis for large B-cell lymphoma and stressed that DA-EPOCH-R showed an excellent outcome with a tolerable toxicity profile in high-risk large B-cell lymphoma patients. We also found that the DA-EPOCH-R group had a lower myelosuppression risk than the R-HyperCVAD, suggesting that DA-EPOCH-R was relatively safe and well-tolerated. The DA-EPOCH-R/MA regimen, based on DA-EPOCH-R, initially exhibited satisfactory five-year OS and PFS outcomes, indicating that high-dose methotrexate and cytarabine can consolidate the efficacy of DA-EPOCH-R to some extent.

CNS relapse is a common and devastating complication of PB-DHL, and the CNS relapse rate is much higher than that of PBL-DLBCL [26]. To date, the underlying CNS progression mechanism is unknown, and prophylactic strategies have become an integral part of current treatment protocols [27]. Common prophylactic CNS strategies for PB-DHL included intrathecal chemoprophylaxis and systemic CNS penetrants such as methotrexate [28]. Depending on previous research, CNS relapse mainly involves the brain parenchyma, and leptomeningeal involvement is rare. Thus, intrathecal chemoprophylaxis, which does not adequately penetrate the brain parenchyma, insufficiently prevents parenchymal CNS recurrence [28, 29]. Holte et al. [30] conducted a study of 156 eligible patients with aggressive B-cell lymphomas and implemented a high-dose of cytarabine plus methotrexate regimen (MA regimen) to reduce the incidence of CNS-related events. The results showed a satisfactory CNS relapse rate of 4.5 %, which is much lower than that in other published reports. Considering that high-dose methotrexate and cytarabine can penetrate the blood-brain barrier, the risk of CNS progression could be further reduced [31, 32]. The MA regimen was expected to reduce the risk of CNS progression, but, in this series, the MA regimen was not superior for CNS prophylaxis in PB-DHL. As a result, the prevention of CNS progression depending on chemotherapy alone was insufficient. In contrast, CNS progression was not detected after ASCT, and more durable remission was achieved in patients who received ASCT than in those who did not (P < 0.001). Considering the fatal outcome of CNS relapse and the limited efficacy of high-dose chemotherapy, ASCT is strongly recommended to reduce the CNS relapse rate and prolong survival.

The contralateral breast tissue is the second most common recurrence site, except in the CNS [18]. This study’s results indicated that breast radiotherapy was better for local control. The survival and relapse rates of patients who received concurrent chemotherapy or post-chemotherapy breast irradiation did not differ. Thus, the breast irradiation sequence of breast irradiation and chemotherapy remains ambiguous in this study. Breast irradiation produced durable remission, justifying its consideration in treating young patients with PB-DHL. If contralateral breast relapse occurred, the clinical outcomes were dismal with unsatisfactory salvage therapies [12]. Therefore, for high-risk young patients, the first-line treatment of PB-DHL patients could be to apply ASCT, despite ASCT not being the first-line treatment for lymphoma.

Since high-intensive chemotherapy alone is insufficient to induce long-term disease control, ASCT could be the most appropriate treatment method if CR or PR is attained, especially for high-risk young patients. ASCT may overcome the poor prognostic implications of this kind of aggressive disease subtype, and there is no doubt that young patients with excellent performance statuses and few comorbidities are the best candidates for ASCT. In this multicentre study, patients who received ASCT had significantly superior outcomes compared to those who did not, suggesting that ASCT greatly reduced the disease progression risk. Similarly, Kim et al. [33] explored a high dose of chemotherapy combined with ASCT for high-risk DLBCL patients and found that ASCT yielded superior OS and PFS. The three-year OS in the ASCT group was 85.0% and was 75.8% in the non-ASCT group (P = 0.038). Further, the three-year PFS was also better in the ASCT group (76.6% vs. 63.3 %; P = 0.007). To date, the ASCT status has not been challenged by high-intensity chemotherapy or novel targeted agents, and it remains an appropriate choice to abrogate the negative prognostic impact of PB-DHL [34]. High-dose chemotherapy followed by ASCT in an upfront setting currently remains the second-line treatment for DLBCL [35], and ASCT is the only feasible therapy offering a cure. Due to the limited efficacy of conventional chemotherapy, dynamic dose-adjusted chemotherapy supported by ASCT should be administered to young PB-DBL patients [36, 37]. The ASCT results showed excellent efficacy and durable remission among patients with CR in a pre-transplantation setting, which is an important initial step for developing ASCT as a first-line treatment strategy for highly aggressive PB-DHL. To further assess the long-term prognosis, a larger-scale investigation that includes more patients receiving ASCT is should be conducted in the future.

TP53 mutations have been shown to be significantly associated with poor overall survival in DLBCL [38, 39]. Clipson A et al. [40] also found the significant association of TP53 mutation with poor overall survival in DLBCL with MYC translocation. In line with this, four cases with TP53 mutation in this research were found to be more refractory to high-dose of chemotherapy and more likely to relapse than those without TP53 mutation. Therefore, new frontline therapeutic combinations including novel target drugs such as BCL-2 inhibitor have shown promising results [41]. Nonetheless, the number of TP53 mutation cases investigated in this study is small and the distinct impact of TP53 mutation in PB-HGBCL remains to be elucidated.

## Limitations

Major limitations of this study include the relatively small sample size and retrospective nature. The sample size may have limited our ability to detect significant differences. Additionally, follow-up for the evaluation of other complications, including secondary malignancies, was short. Nevertheless, considering the rarity of PB-DHL and the lack of a previously published cohort study, the results of this study still have great guiding significance (Additional file 1).

## Conclusions

This multicentre North-China collaboration provides insights into effective therapeutic management and failure patterns of PB-DHL. DA-EPOCH-R/MA was a promising regimen for PB-DHL, and breast irradiation yielded complementary benefits for relapse reduction. Notably, ASCT significantly decreased disease relapse and provided a potential curative PB-DHL intervention, justifying ASCT as a first-line therapy for young patients. The exploration of more effective treatment strategies for patients with PB-DHL remains promising.

## Supplementary Information

**Additional file 1.** Original data.

## Data Availability

All the original data used in this article is available from the corresponding authors on reasonable request.
